# Draft Genome Sequence of *Escherichia coli* Strain SN137, a Bacterium with Extracellular Proteolytic Activity on Immunoglobulins and Persistence in Human Tissue Blood

**DOI:** 10.1128/genomeA.01455-17

**Published:** 2018-01-18

**Authors:** Salustio Najera-Hernandez, Maria Patricia Sanchez-Alonso, Estela Anastacio-Marcelino, Erasmo Negrete-Abascal, Candelario Vazquez-Cruz

**Affiliations:** a Centro de Investigaciones en Ciencias Microbiológicas, Instituto de Ciencias, Benemérita Universidad Autónoma de Puebla, Puebla, Puebla, Mexico; b Carrera de Biología, Facultad de Estudios Superiores Iztacala, UNAM, Tlalnepantla, Estado de México, Mexico

## Abstract

The draft genome sequence of *Escherichia coli* strain SN137 is reported here. The genome comprises 172 contigs, corresponding to 4.9 Mb with 50% G+C content, and contains several genes related to pathogenicity that explain its survival in human hematic tissue.

## GENOME ANNOUNCEMENT

*Escherichia coli* lineages are important components of the human and animal microbiomes (1–3). This bacterium is a part of human beings their entire life and is indispensable for optimal intestinal function (4–7). However, there are genetic variants that contain virulence genes, so these bacteria can be intestinal pathogens in humans and other mammals (8, 9). Additionally, extraintestinal *E. coli* pathogens have been described and are designated with the acronym ExPEC (extraintestinal pathogenic *E. coli*) (10, 11). The different virulence factors constituting the arsenal of a pathogen, including proteases, inactivating immunoglobulins, and other homeostatic molecules, are important in the microbiome-host homeostasis breakdown (12).

This work reports the genome sequence of an *E. coli* strain (SN137) obtained from a human blood culture that was resistant to four different antimicrobials and that presented high proteolytic activity against bovine serum albumin and human IgG and IgA.

Total genomic DNA from *E. coli* SN137 was obtained by lysozyme digestion and phenol extraction (13). Purified high-molecular-weight DNA was processed to identify the nucleotide sequence in the Laboratorio Nacional para la Genética de la Biodiversidad del CINVESTAV Campus Irapuato, Mexico. Sequence DNA was obtained using GS FLX 454 technology from Roche (Branford, CT, USA) and corroborated by the MiSeq system (Illumina, San Diego, CA, USA).

The genome sequence using 454 technology provided 32-fold coverage. The reads were filtered by quality and assembled with Mira 4 software (14), obtaining 172 contigs that were used to piece together a 4.96-Mb genome. The reconstituted genes indicated that the strain belongs to the D molecular phylogroup. A multilocus sequence type (MLST) analysis of this strain classified it as sequence type 3005 (ST3005) but with variation in the *fumC* marker. From these results, it was concluded that *E. coli* SN137 is an avian pathogenic *E. coli* (APEC) infecting humans. In the genome sequence, we identified the F fertility factor and an important part of the pathogenicity island (GenBank accession number KT777641) described in different *Shigella* sp. strains. Additionally, genes were identified that encode PilA, curli, and Bfp fimbria and encode for resistance to mercury, sulfas, tetracycline, and β-lactam antibiotics, confirming its multiresistance. Type II, III, and VI secretion systems related to virulence and injection of effector molecules into host cells were identified. Moreover, this strain has genes encoding proteases and protease inhibitors, such as alpha-2-macroglobulin, and genes encoding components associated with cell invasion and serum resistance, which explains the capability of *E. coli* SN137 to infect tissue blood.

### Accession number(s).

This whole-genome shotgun project has been deposited at DDBJ/EMBL/GenBank under the accession number NKYO00000000. The version described in this paper is the first version, NKYO01000000.
